# A single latent channel is sufficient for biomedical glottis segmentation

**DOI:** 10.1038/s41598-022-17764-1

**Published:** 2022-08-22

**Authors:** Andreas M. Kist, Katharina Breininger, Marion Dörrich, Stephan Dürr, Anne Schützenberger, Marion Semmler

**Affiliations:** 1 Department Artificial Intelligence in Biomedical Engineering, Friedrich-Alexander-University Erlangen-Nürnberg, 91052 Erlangen, Germany; 2 Division of Phoniatrics and Pediatric Audiology at the Department of Otorhinolaryngology, Head and Neck Surgery, University Hospital Erlangen, Friedrich-Alexander-University Erlangen-Nürnberg, 91054 Erlangen, Germany

**Keywords:** Medical imaging, Biomedical engineering

## Abstract

Glottis segmentation is a crucial step to quantify endoscopic footage in laryngeal high-speed videoendoscopy. Recent advances in deep neural networks for glottis segmentation allow for a fully automatic workflow. However, exact knowledge of integral parts of these deep segmentation networks remains unknown, and understanding the inner workings is crucial for acceptance in clinical practice. Here, we show that a single latent channel as a bottleneck layer is sufficient for glottal area segmentation using systematic ablations. We further demonstrate that the latent space is an abstraction of the glottal area segmentation relying on three spatially defined pixel subtypes allowing for a transparent interpretation. We further provide evidence that the latent space is highly correlated with the glottal area waveform, can be encoded with four bits, and decoded using lean decoders while maintaining a high reconstruction accuracy. Our findings suggest that glottis segmentation is a task that can be highly optimized to gain very efficient and explainable deep neural networks, important for application in the clinic. In the future, we believe that online deep learning-assisted monitoring is a game-changer in laryngeal examinations.

## Introduction

A functional voice is a crucial factor for a successful social embedding. To determine functionality, voice physiology is commonly *qualitatively* assessed using stroboscopy^1^. Laryngeal high-speed videoendoscopy (HSV) is an alternative, currently emerging technique, that also allows *quantification* of voice physiology^2^. With HSV, the vocal fold motion is typically recorded at several thousand frames per second, therefore visualizing each glottal cycle accurately^3^. The glottis or glottal area, the opening between the vocal folds, is a good proxy for the cyclic behavior of the vocal fold oscillations and is of major interest for quantitative data analysis.

The glottis can be segmented using several image analysis techniques^4^, among others active contours^5^, Gabor filters^6^ and thresholding combined with level set methods^7^. Recently, deep neural networks for semantic segmentation have been utilized for glottis segmentation^8,9^. Additionally, optimized deep neural networks for clinical applicability have been proposed^10^. However, these deep neural networks commonly have a black box character, lowering their acceptance in a clinical environment^11^. This effect can be typically reduced when providing insights into the inner workings of a proposed algorithm. Despite the fact that we know that deep neural networks are well suited for the task of glottis segmentation, we are lacking a fundamental understanding of what are the necessities and learned representations of these deep neural networks.

Autoencoders or, in general, encoder–decoder architectures consist of contraction and expansion paths^12,13^, where the bottleneck layer is referred to as code layer or latent space. The latent space is thought to contain a high-level embedding of the raw input image. The inspection of this latent space is highly interesting for generative adversarial networks (GANs), as the latent space can be used in GANs to specifically direct the generative image to enable face editing^14,15^, image embedding interpolation^16^ and novelty detection^17^. For semantic segmentation, the latent space has also been shown beneficial in multi-task architectures^18,19^. However, little is known about what the latent space represents in biomedical image analysis.

In this work, we are characterizing the latent space, a higher-order representation of the endoscopic image, embedded in a semantic segmentation architecture. We systematically investigate its properties and how alterations to the latent space result in differences in the glottis segmentation prediction. With this, we will leverage the potential of latent space information in a clinical context in two ways: explainability and computation-efficient networks.

## Materials and methods

### Data and preprocessing

To train and evaluate deep neural networks for glottis segmentation, we used the open Benchmark for Automatic Glottis Segmentation (BAGLS,^9^). We used the full training and test dataset containing 55,750 and 3,500 endoscopic images and their segmentation mask, respectively. All subsequent experiments were carried out in accordance with the relevant guidelines and regulations. For training, we resized all images to 512$\times$256 px, which is the native resolution of most images in the dataset. For validation, we used 10% randomly selected frames from the training dataset. All endoscopic images were converted to grayscale. The input image intensities were normalized to $-1$ and 1, the segmentation masks were normalized to 0 and 1, where 0 is the background and 1 is the glottal area. We randomly applied data augmentation to the training data using Gaussian blur (kernel size between 3 and 7 with a $\sigma$ ranging from 0.8 to 1.4), rotation ($-30$ to $30^{\circ }$), horizontal flip and gamma adjustments (random gamma between 0.8 and 1.2). We also use short video snippets of 30 frames available in the BAGLS dataset for time-variant data analysis (Fig. 3). Videos are processed as single frames on a single frame basis.

#### Glottal area waveform (GAW)

The glottal area waveform (GAW) is a one-dimensional representation of the vocal fold oscillation behavior. Each time point of the GAW is computed as the sum of foreground pixels in the glottal area segmentation mask at the given time point^20^.

### Deep neural networks

#### Architecture

**Figure 1:**
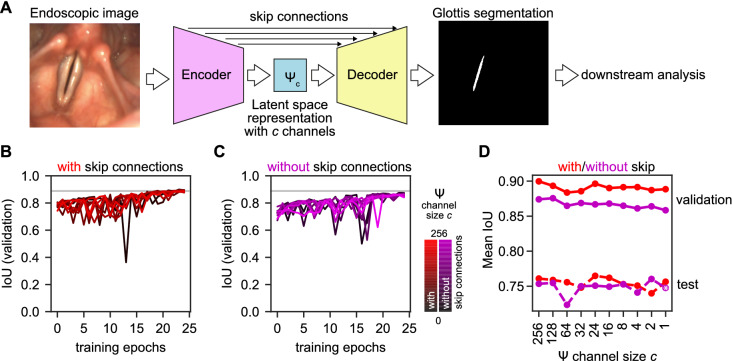
A single latent space channel is sufficient for glottis segmentation. (**A**) Glottis segmentation of endoscopic images using deep neural networks (DNNs) with latent space $\Psi$. (**B**) Convergence of segmentation DNNs across different latent space channels with enabled skip connections. Gradient from black to red indicates fewer channels. The gray line indicates maximum IoU score. (**C**) Convergence of segmentation DNNs across different latent space channels with disabled skip connections. Gradient from black to magenta indicates fewer channels. The gray line indicates maximum IoU score from panel **B**. (**D**) Performance of best performing segmentation DNNs on validation set (solid lines) and evaluated on test set (dashed lines) with enabled (with, red) and disabled (without, magenta) skip connections across latent space ($\Psi$) channels measured by mean intersection over union (IoU). The asterisk indicates the architecture used in the subsequent experiments.

The baseline glottis segmentation network is based on the U-Net architecture^21^ modified as described in^10^. Briefly, we rely on an encoder–decoder architecture to change the image domain from endoscopic image to glottal area segmentation (see Fig. 1). Initially, we use skip connections between encoder and decoder to pass mid-level information by concatenation. We set up deep neural networks in TensorFlow 2.6 using the Keras high-level package. All experiments were performed on an NVIDIA RTX 3090. We trained for 25 epochs at a constant learning rate of $10^{-4}$ using the Adam optimizer^22^, which has been shown as a successful strategy previously^9^. Each convolutional layer used a kernel size of 3$\times$3 and $f_L$ convolutional filters that follow Eq. (1):

1 $$\begin{aligned} f_L = f_{base} \cdot 2^{d}, \end{aligned}$$

where $f_L$ is the number of convolutional filters which equals the number of channels in a given layer. At a given network depth *d* (in our baseline model $d \in \{0,1,2,3,4\}$) and a given initial base filter size $f_{base}$ ($f_{base}=16$ in our baseline model) we gain a total of $f_L=256$ latent space channels *c* at maximum network depth $d=4$. After each convolution layer, we applied batch normalization^23^. After batch normalization, we used the ReLU function as non-linearity (Eq. 2). However, in the latent space $\Psi$ (Fig. 1A) we used the ReLU6 function (Eq. 3) that is clipped between 0 and 6. The ReLU6 function has been shown to be a good choice for low-bit quantization^24,25^ and to foster learning early sparse features^26^.

2 $$\begin{aligned}&\text{ ReLU }(x) = \max (0, x) \end{aligned}$$

3 $$\begin{aligned}&\text{ ReLU6 }(x) = \min (\max (0, x), 6). \end{aligned}$$

During training, we were minimizing the Dice loss^27^ as defined in Eq. (4) by comparing the predicted glottis segmentation mask $\hat{\mathbf{y}}$ to the ground-truth segmentation mask $\mathbf{y}$.

4 $$\begin{aligned} \text{ Dice }(\mathbf{y},\hat{\mathbf{y}})=1-\frac{2\mathbf{y}\hat{\mathbf{y}}+1}{\mathbf{y}+\hat{\mathbf{y}}+1}. \end{aligned}$$

#### Latent space $\Psi$

The latent space $\Psi$ is a high-level representation of the initial endoscopy image at the end of the encoder and serves as input to the decoder (Fig. 1). It can be interpreted as an image with $f_L$ “color” channels. For latent space investigations, we changed $f_L$ from its initial value (here: 256), as defined by Eq. (1), to a fixed value ranging from 1 to $f_L$. When $f_L=1$, we refer to the latent space as latent space image $\Psi _1$. For all experiments after Fig. 1, we are using the latent space images $\Psi _1$ generated by the architecture without skip connections and a single latent space image, indicated in Fig. 1D with an asterisk.

#### Decoder experiments

The initial decoder is constructed as described in the section *Architecture*. For decoder experiments, we used different strategies to construct the decoder (Fig. 5A). The latent space image $\Psi _1$ is used as the sole input to the decoder with a resolution of $32\times 16\times 1$ (height $\times$ width $\times$ channels). Next, we used a combination of 2D upsampling operations (Upsampling2D) operations and either one or two convolutional layers with $f_D$ channels, where $f_D \in \{1, 2, 4, 8\}$. For each Upsampling2D-Convolution cycle $UC \in \{1, 2, 4\}$, the Upsampling2D operation uses a scaling factor $s \in \{16, 8, 2\}$, respectively, to ensure a full upsampling to the original image resolution (512$\times$256 px). For training, we converted each training image to its latent space representation by using the final model used in latent space data analysis (result of Fig. 1D, with no skip connections and $f_L=1$). We converted each latent space image in uint8 as we have shown that eight-bit are sufficient for high-level encoding (Fig. 4A, B).

#### Class activation maps

To highlight relevant features in the neural network architecture, class activation maps (CAMs) were used^28^. We relied on an adaptation of the Grad-CAM method^29^ for semantic segmentation architectures (Seg-Grad-CAM^30^).

We generated a heatmap $\mathbf{H}_{\mathbf{l}}^{\mathbf{c}}$ at layer *l* for either glottis segmentation ($c=1$) or background ($c=0$) using a rectified, weighted sum of the feature maps *A* across kernels $k \in [1,K]$ at given layer *l*:

5 $$\begin{aligned} \mathbf{H}_{\mathbf{l}}^{\mathbf{c}} = \text {ReLU} \left( \sum _{k=1} \alpha ^c_k \mathbf{A}_k\right) , \end{aligned}$$

where the weight of each gradient activation map $\alpha ^c_k$ for each kernel *k* and given class *c* for all pixels *N* in a given image, indexed by *u* and *v*, is defined as follows:

6 $$\begin{aligned} \alpha _k = \frac{1}{N} \sum _{u,v} \frac{\partial y^c}{\partial \mathbf{A}^k_{uv}}. \end{aligned}$$

$y^c$ is defined as the logit for a given class *c*.

### Bit encoding

To determine the information content carried by the latent space, we reduced the encoding to a fixed bit encoding. We created a histogram of a given latent space image and divided it into $2^{bits}$ bins. We then set each pixel in a given bin range to the average value in a given bin (Fig. 4C). The resulting new latent space image is provided to the decoder and the reconstructed image is compared to the ground-truth segmentation mask. We used the mean squared error (MSE) and the intersection over union (IoU) score (see *Evaluation*) as evaluation metrics.

### Evaluation

We evaluated the segmentation quality using the IoU (intersection over union) score^31^ as defined in Eq. (7).

7 $$\begin{aligned} \text{ IoU }(\mathbf{y}, \hat{\mathbf{y}}) = \frac{\mathbf{y} \cap \hat{\mathbf{y}}}{\mathbf{y} \cup \hat{\mathbf{y}}}. \end{aligned}$$

We further computed the correlation between the latent space image $\Psi _1$ across time (in the equation refered as $\mathbf{x}$) and the GAW ($\mathbf{y}$) as follows:

8 $$\begin{aligned} \text{ r}_{xy} = \frac{\sum _{i=1}^{n} (x_i-\bar{x})(y_i-\bar{y})}{(n-1) \cdot s_x \cdot s_y}, \end{aligned}$$

where *n* is the number of time points/samples, $\bar{x}$ and $\bar{y}$ the average of $\mathbf{x}$ and $\mathbf{y}$, respectively, and $s_x$ and $s_y$ are the sample standard deviation of $\mathbf{x}$ and $\mathbf{y}$, respectively.

## Results

### A single latent space channel is sufficient for glottis segmentation

To understand which components are crucial in a segmentation deep neural network, we performed an ablation study on a modified U-Net architecture (see “Materials and methods”). We trained a full-size, complete U-Net to perform glottis segmentation (Fig. 1A), similar to the previous works^9,10^. The latent space $\Psi$, the ultimate bottleneck that connects encoder and decoder in the full U-Net, has initially 1024 channels (Fig. 1A), when 64 filters are used in the first layer ($f_{base}=64$). In this work, we use a reference implementation with 16 filters in the first layer ($f_{base}=16$) and thus, 256 channels in the latent space, as this has been shown previously to provide comparable performance compared to $f_L=1024$^10^.

We systematically reduced the amount of channels in the latent space to determine the minimum viable latent space. We found that even a single latent space channel is sufficient to encode the glottal area segmentation (Fig. 1B). However, we hypothesized that the skip connections in the U-Net allow rescuing the strong limitation in the bottleneck. Hence, we removed the skip connections and found that the segmentation accuracy in the validation set was reduced across configurations (Fig. 1C). However, the network architecture is still able to provide accurate glottis segmentations (Fig. 1D), and has a performance on the test set similar to higher latent space encodings and enabled skip connections. The general drop in performance is due to the nature of the test set that contains a balanced blend of data across multiple hospitals with a variety in data quality, whereas this is not granted in the training dataset.

To further investigate of the reconstruction capabilities of the ablated architecture, we evaluated its autoencoder abilities. In Supplementary Figure 1, we show that the autoencoder ability across latent space $\Psi$ channels are perceptually uniform. However, the disabling the skip connections had a major effect on the reconstruction, and high-frequency details were lost. Notably, the glottal area seemed to be largely retained which is in line with our findings related to glottis segmentation.

To avoid any effect of the dataset itself on the study, we systematically ablated the training dataset, while evaluating on the BAGLS test dataset. We found that our results hold across dataset sizes (Supplementary Figure 2). While the validation IoU reaches a similar, but slightly declining score across dataset sizes (Supplementary Figure 2A), we found that the performance on the test set is stable across dataset sizes (Supplementary Figure 2B).

In summary, we show that a single latent space channel is sufficient for glottis segmentation. We will refer to this single latent space channel as latent space image $\Psi _1$, and use the architecture shown in Fig. 1D (indicated with an asterisk) for subsequent experiments.

### The latent space encodes glottis location and shape

**Figure 2:**
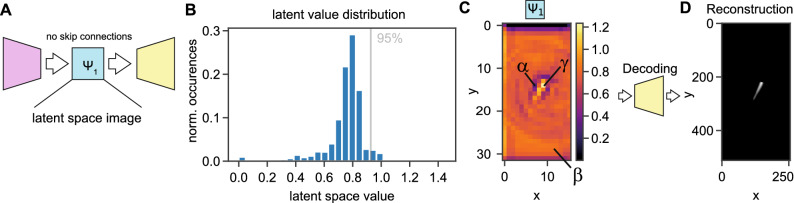
The latent space image $\Psi _1$ values provide interpretable context. (**A**) Deep neural network (DNN) used for further experiments. DNN consists only of a single latent space image $\Psi _1$ without skip connections. (**B**) Value distribution of the latent space image $\Psi _1$ across all pixels of all images in the BAGLS training dataset. We indicated the 95% confidence interval that is used for defining $\gamma$ pixels. (**C**) The average latent space image $\Psi _1$ across 30 frames. We indicate the three pixel subtypes, $\alpha$ for glottis refining, $\beta$ for background-defining, and $\gamma$ for glottal area defining pixels. (**D**) The average reconstruction obtained from feeding $\Psi _1$ from panel **C** into the decoder.

Next, we investigated the properties of the latent space (Fig. 2A). We encoded all images of the BAGLS training dataset to gain a collection of latent space $\Psi$ images. We first determined if any single pixel is directly correlated for with the glottal area. We found that the correlation values follow a normal-like distribution centered around 0.00 with a standard deviation of 0.12 (Supplementary Figure 3). We then investigated the value distribution of the latent space. The histogram shows a distribution centered around 0.8 ($\hbox {mean}=0.75$, $\hbox {median}=0.78$, $\hbox {mode}=0.80$), with a long tail towards 0 and a very short tail above 0.8 (Fig. 2B). Interestingly, we clipped the available value space in the latent space between 0 and 6 (see “Materials and methods”), however, the largest value we observed was 1.45, indicating that we were not constrained by our activation function.

To understand the meaning behind these values, we found that values around 0.8 encoded for background (referred to as $\beta$ pixels), values higher than 0.8 (especially higher than the 95% percentile) defined the glottal area (hereafter referred to as $\gamma$), and values lower of 0.8 were glottal area shaping values ($\alpha$ pixels, Fig. 2C). More examples are shown in Supplementary Figure 4. Further, the latent space image $\Psi _1$ is encoding the spatial location of the glottal area in *x* and *y*. We confirmed this by generating an artificial latent space image $\Psi _1$ and varying *x* and *y* location, $\gamma$ pixel value intensity and pixel drawing radius (Supplementary Movie 1). Values higher than 1.5 for $\gamma$ resulted in image artefacts and were also not found in the general latent value distribution (Fig. 2B). We further investigated the role of the value drop (values $< 0.8$) for $\alpha$ pixels adjacent to the glottis-defining $\beta$ pixels (values $> 0.8$). Our results suggest that $\alpha$ pixels in the surrounding of $\gamma$ pixels are shaping the glottal area’s extent and refining its appearance (Supplementary Movie 2). Taken together, the interplay between $\alpha$ and $\gamma$ pixels is crucial for an accurate glottis segmentation, to gain an accurate reconstruction as shown in Fig. 2D.

To better understand the encoding and decoding properties of the architecture, we were analyzing class activation maps (CAMs). As shown in Supplementary Figure 5, we found that during encoding the background and the foreground, i.e. the glottal area, get well separated and merge in the single latent space image $\Psi _1$. In the decoding layers, the background class focused on the glottal area outlines, whereas the foreground glottal area was constantly refined. In agreement with our previous findings, the CAMs suggest that $\Psi _1$ is a powerful and sufficient representation of the glottal area.

### Thresholded latent space is highly correlated with the glottal area waveform

**Figure 3:**
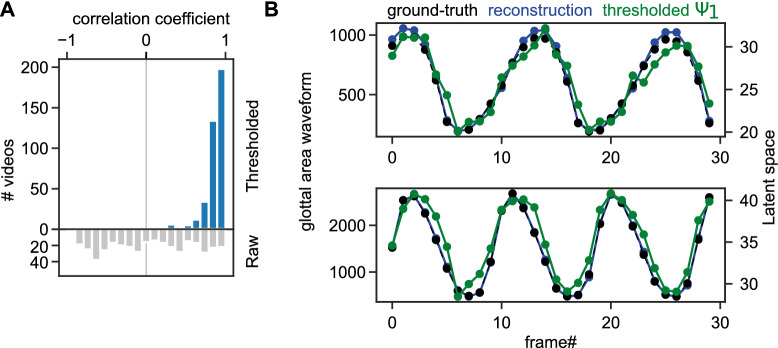
Thresholded latent space image $\Psi _1$ is highly correlated with glottal area waveform. (**A**) Distribution of correlation values across videos using either the raw latent space image $\Psi _1$ or the thresholded $\Psi _1$ using the 95% confidence interval as threshold (see Fig. 2A). Correlation was computed from every latent space to glottal area waveform of 30 frames ($\hbox {N}=399$ videos). (**B**) Two exemplary videos showing the original ground-truth glottal area waveform (GAW, black), the segmentation prediction of the deep neural network with a single latent channel (blue) and the thresholded latent space image $\Psi _1$ from the same network (green).

The glottal area waveform (GAW) is a time-variant signal important for assessing vocal fold physiology^2,32^. We, therefore, asked if the latent space image $\Psi _1$ is a good proxy for the GAW. To answer this question, we used short video fragments from the BAGLS dataset and converted the provided ground truth segmentation mask to the GAW (see “Materials and methods”). We followed two approaches: (1) summing all values in $\Psi _1$ and (2) threshold $\Psi$ at 95% confidence interval ($\hbox {value} = 0.8$) and then summing the positive pixels. In Fig. 3A, we show that approach (1) is correlated to a limited extent with GAWs (on average $0.03 \pm 0.56$), however, approach (2) is highly correlated with the GAW, on average $0.84 \pm 0.18$. Figure 3B shows two exemplary videos with corresponding ground-truth GAW, the GAW generated by using the segmentation masks reconstructed by the decoder, and the thresholded $\Psi _1$ waveform.

### A low bit encoding is sufficient for glottis reconstruction

**Figure 4:**
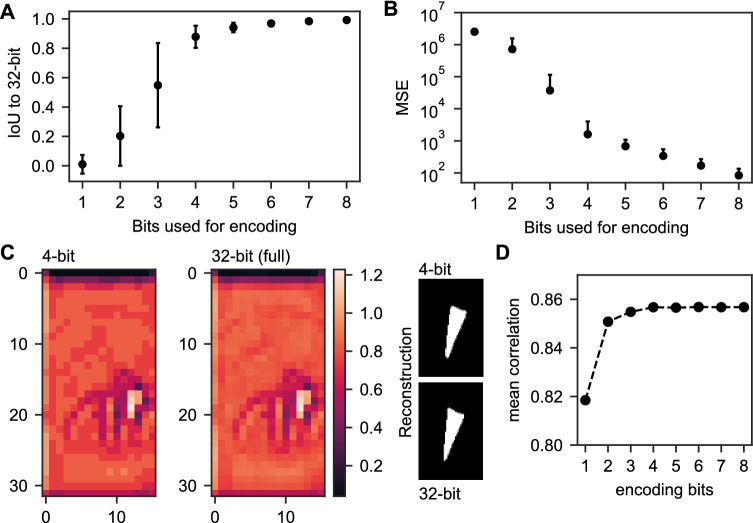
Four bits were sufficient for accurate reconstruction. (**A**) IoU score comparing the reconstructions from lower bit encodings to the full 32-bit reconstruction. (**B**) Mean squared error (MSE) for lower bit reconstruction and full 32-bit reconstruction. (**C**) Example for 4-bit and respective 32-bit encoding with respective reconstruction. (**D**) Mean correlation of low bit latent space image $\Psi _1$ and glottal area waveform (GAW).

As the value range is very limited in the latent space (Fig. 2B) and the existence of $\alpha$, $\beta$, and $\gamma$ pixels, we hypothesized that a low bit depth is sufficient for encoding the latent space $\Psi _1$ for accurate glottal area reconstruction. By reducing the bit depth from 32-bit floating point to a range of 1 to 8-bit, we found that 4-bit encoding is sufficient for high-quality reconstructions (Fig. 4A–C). Specifically, with 4-bit encoding the IoU score became stable and showed a low error, which is neglectable with 8-bit encoding (Fig. 4A). The mean-squared error (MSE) between full 32-bit reconstruction and low-bit reconstruction declined as expected with increasing bit depth, but in terms of absolute values, we observed some deviation from the original reconstruction (Fig. 4B). We further were able to reproduce the high correlation of $\Psi _1$ with the glottal area waveform (Fig. 4D). In summary, we showed that 4-bit encoding is sufficient for subjectively similar glottis segmentations compared to full 32-bit encoding.

### Light-weight decoders are capable of reconstructing the glottal area

**Figure 5:**
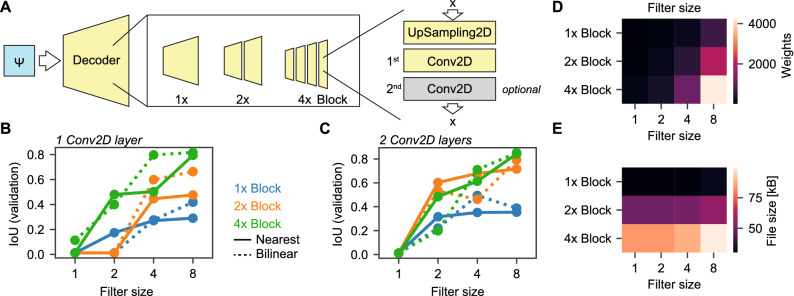
Lean decoders are sufficient for glottis reconstruction from a single latent space image $\Psi _1$. (**A**) Decoding from latent space image $\Psi _1$. Evaluated decoders consist of either 1, 2 or 4 upsampling-convolution blocks (right panel), wherein one or two convolutional layers can be present. The filter size of each convolutional layer was fixed (see panels **B–E**). (**B**) IoU scores of different decoder blocks (color-coded, 1 blue, 2 orange, 4 green) using either nearest neighbor (solid lines) or bilinear upsampling (dashed lines). (**C**): Same as panel **B**, but with two convolutional layers. (**D**) Trainable weights across decoder settings for two convolutional layers per block. (**E**) File size across decoder settings for two convolutional layers per block.

As the latent space image $\Psi _1$ was easily interpretable and showed a low-level complexity, we hypothesized that the decoder architecture can be largely simplified. Hence, we investigated how many convolutional filters and how many upsampling steps are necessary for decoding from the single latent space image $\Psi _1$ introduced before (Fig. 5A). Further, we were interested if the upsampling strategy (nearest neighbours vs. bilinear interpolation) and multiple convolutional layers would affect the decoding ability (Fig. 5A). When using a single convolutional layer in each upsampling step, we found that one and two convolutional filters were not sufficient for decoding and that four convolutional filters were only sufficient in a single configuration (4x upsampling and bilinear interpolation) as shown in Fig. 5B. The best results were achieved using eight convolutional filters together with 4x upsampling, which resulted in decent IoU scores (0.817, Fig. 5B). Using two convolutional layers in each upsampling step, however, allowed 2x upsampling being competitive in the eight convolutional filters configuration. In general, two and four convolutional filters showed better performance compared to the single convolutional layer experiment. However, these were not competitive with the configurations showing eight convolutional filters (Fig. 5C). The top performance with two convolutional layers per block, eight convolutional filters and 4x upsampling with IoU=0.852 was slightly outperforming the single convolutional layer configurations. Despite the higher amount of trainable parameters in this configuration (Fig. 5D), it had a relatively stable file size of 99 kB (Fig. 5E). It is astonishing that even configurations with less than 200 trainable parameters achieved IoU scores higher than 0.4 (Table 1).

**Table 1:** Overview of all decoder configurations tested.

Blocks	Filter size	Conv2D layer	Weights	File size (kB)	IoU (nearest)	IoU (bilinear)
1x	1	One	12	19	0.01	0.01
	2		24	19	0.01	0.17
	4		48	19	0.28	0.27
	8		96	19	0.42	0.29
2x	1		23	31	0.01	0.01
	2		64	31	0.01	0.01
	4		200	31	0.60	0.45
	8		688	34	0.66	0.48
4x	1		45	51	0.11	0.01
	2		144	51	0.40	0.48
	4		504	53	0.80	0.50
	8		1872	58	0.82	0.79
1x	1	Two	23	30	0.01	0.01
	2		64	30	0.22	0.32
	4		200	30	0.49	0.35
	8		688	33	0.39	0.35
2x	1		45	49	0.01	0.01
	2		144	49	0.53	0.60
	4		504	51	0.46	0.68
	8		1872	55	0.79	0.72
4x	1		89	84	0.01	0.01
	2		304	84	0.20	0.48
	4		1112	88	0.71	0.61
	8		4240	100	**0.85**	**0.84**

Significant values are in bold.

## Discussion

In this work, we found that a single channel in the latent space of an encoder–decoder architecture was sufficient for glottal area reconstruction. We further showed that the latent space forms an image that has interpretable properties, such as background ($\beta$), glottal area defining ($\gamma$) and refining ($\alpha$) pixels. Our findings suggested that encoder–decoder frameworks are not only suitable for glottis segmentation, but also provide a higher-order approximation of the glottal area sufficiently encoded in a significantly smaller, single channel image. Together with a low bit encoding (Fig. 4), it may serve as an efficient data storage system for glottis segmentations, which would be important for a variety of downstream analyses to compute quantitative parameters. The latent space image was easily reconstructed using efficient decoders as presented in Fig. 5.

Our results highlighted that by mining the deep neural network crucial for a clinical task, in this case glottis segmentation, we were able to truly determine what the network has learned, and were able to interpret these results (Fig. 1). We also were able to show what effect alterations have on the latent channel (Supplementary Movies 1 and 2). Current state-of-the-art explainable AI methods, such as class activation maps, are highly under debate^33,34^, whereas our approach is easy interpretable and transparent, despite the fact CAMs in this setting seemed to be in agreement with our findings (Supplementary Figure 5). This transparency also allows in principle to better understand failure cases, as they can directly be investigated, reported and corrected.

Our findings showed that the reduced latent space provides a high-level representation of the segmentation. However, in this study we did not directly investigate how the high-level representation disentagles semantic information, such as glottis shape, glottis opening or pathologies. Disentanglement analysis^35^, especially in the field of medical analysis, would help to better understand what the network learns and would allow multiple possible and plausible results. Modifications to the neural architecture, such as the use of appropriate loss functions that support disentanglement^36^ or incorporating priors^37^, should be investigated in the future.

In this study, we particularly focused on the latent space and its minimal extent for glottis segmentation. We found that removing the U-Net-specific skip connections yielded lower IoU scores in the validation set, whereas we did not find any differences in the test set, i.e. on independent, unseen data (Fig. 1D). This is in line with a previous study^10^, where the authors showed that the kind of skip connection is not important (adding or concatenating channels from encoder to decoder) for glottis segmentation. They also found a significant drop in the validation IoU score, when removing the skip connections. However, they have not specifically investigated the role of skip connections in this context. It remains elusive if certain data other than laryngeal endoscopy images benefit from enabled skip connections for segmentation tasks. Notably, adding further processing layers to the skip connections does improve the performance on medical data^38^.

Glottis segmentation is a straight forward task and was previously approached using variations of thresholding-based techniques^7,20,39^. Therefore, it is likely that the encoder–decoder architecture would learn a smart and non-linear thresholding algorithm. However, other modalities, such as anterior-posterior point prediction for midline estimation^19^ and vocal fold localization for paralysis analysis^40^ may not benefit from this very constrained latent space. Future studies should address these limitations and speculate about the necessity of an increased latent space crucial for multitask architectures, as the latent space has been shown useful for midline estimation^19^.

The U-Net is a very powerful starting point for biomedical image segmentation tasks, also for glottis segmentation^4,9,41^. Modifications to this architecture, such as convolutional layers with LSTM memory cells^42^ as shown by^41^ may improve the glottal segmentation accuracy. Minimizing the parameter space was also shown beneficial in glottis segmentation^10^ and across biomedical image analysis tasks^43^. Also, more sophisticated encoding backbones, such as the ResNet^44^ and the EfficientNet^45^ architecture showed superior performance in glottis segmentation, especially on more dissimilar data sources^20^. Future research should investigate, if these architectures are able to detect and encode better high-level features in the latent space, such that a potentially higher dimensionality in the latent space yields further performance improvements.

## Conclusion

With this work, we contributed to the understanding of how glottis segmentation is performed by deep neural networks and that we were able to uncover the deep neural network black box character by identifying three value ranges with a specific role, namely $\alpha$, $\beta$ and $\gamma$ pixels. Future studies may elucidate if these three subclasses can be further refined and if they occur across architectures and segmentation tasks. In general, our findings would allow very efficient architectures leveraging the potential of real-time applications of glottis segmentations in a clinical setting and maybe used together with recent advances in HSV systems^46^. Further research on quantitative measures may include how the latent space image $\Psi$ influences these computations and if the latent space is also sufficient for approximating complex quantitative parameters to assess easily voice physiology.

## Supplementary Information

Supplementary Information 1.

Supplementary Information 2.

Supplementary Information 3.

## Data Availability

We provide all relevant code at https://github.com/ankilab/latent. In this study, we relied on the open BAGLS dataset. We provide all latent space images for decoder training and the used model for latent space image analysis at https://zenodo.org/record/5772799.
