# Supplementary material for: A single latent channel is sufficient for biomedical glottis segmentation
Source: Sci Rep. 2022 Aug 22;12:14292. doi: 10.1038/s41598-022-17764-1 (PMC9395348; doi:10.1038/s41598-022-17764-1)
Supplement: Supplementary file 1 — Supplementary Information 1. [file 41598_2022_17764_MOESM1_ESM.pdf]

A single latent channel is sufficient for biomedical glottis segmentation

Andreas M. Kist, Katharina Breininger, Marion Dörrich, Stephan Dürr, Anne Schützenberger, Marion Semmler

Supplementary Information

S1 Fig. Reconstruction of input images using ablated latent space

S2 Fig. Segmentation performance on unseen data is stable across training dataset sizes

S3 Fig. Latent space pixels are not directly correlated with the glottal area.

S4 Fig. Latent space coding is consistent across recordings.

S5 Fig. Class activation maps show how the latent space is related to the input image.

S1 Video. γ pixels define the glottal area.

The glottal area is defined in a range between 0.8 and 1 leading to a nice segmentation.

Values higher than 1 tend to create artifacts.

S2 Video. α pixels shape glottal area.

This movie shows the interplay between α and γ pixels.

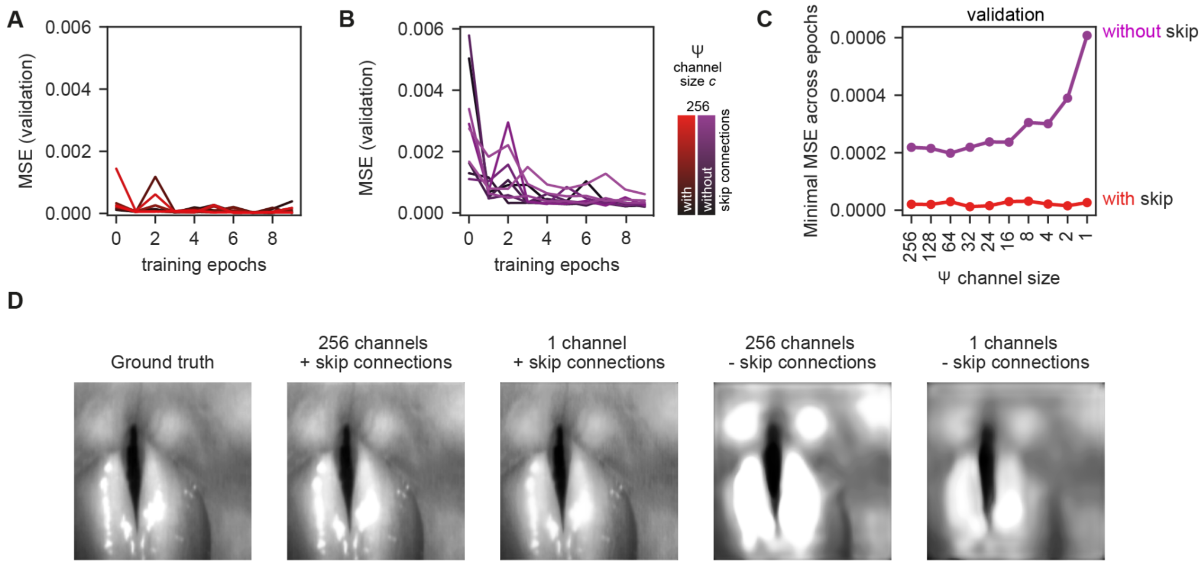

Supplementary Figure S1. Reconstruction of input images using ablated latent space. A: Validation mean squared error (MSE) loss across training epochs for neural architectures across latent space channel restriction (256 to 1 latent channel), color-coded. B: Same as panel A, but without skip connections. C: Best validation loss across epochs, color-coded for with (red) and without (magenta) skip connections. D: Example reconstructions for a given input image for 256 or 1 and with or without skip connections. Note the good reconstruction of the glottal area.

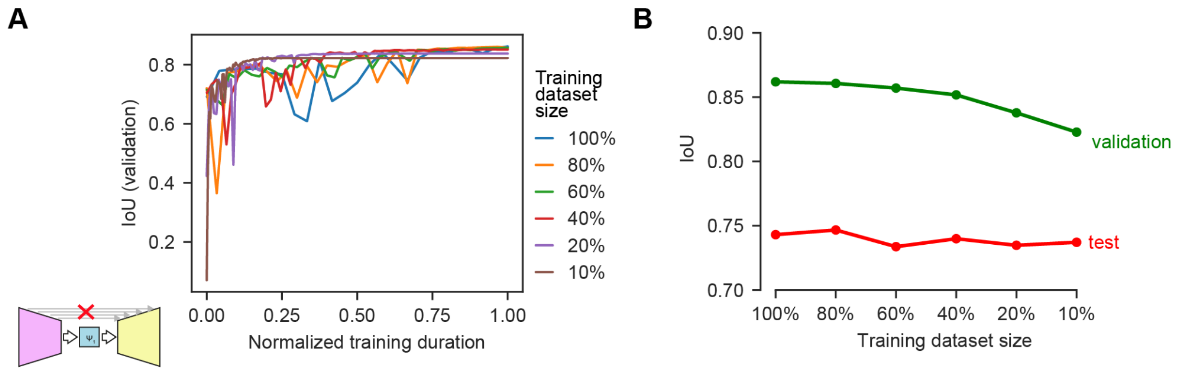

Supplementary Figure S2. Segmentation performance on unseen data is stable across training dataset sizes. The neural architecture with the latent space image Ψ_1 and without skip connections was used. A: Normalized training duration for different dataset sizes. Training duration was adjusted depending on the data amount, such that the same amount of images were shown during training. B: Achieved IoU on the validation (green) and on the test (red) dataset across training dataset sizes. For the validation set, the maximum IoU across training epochs is reported. For the test set, the average IoU across test images is reported.

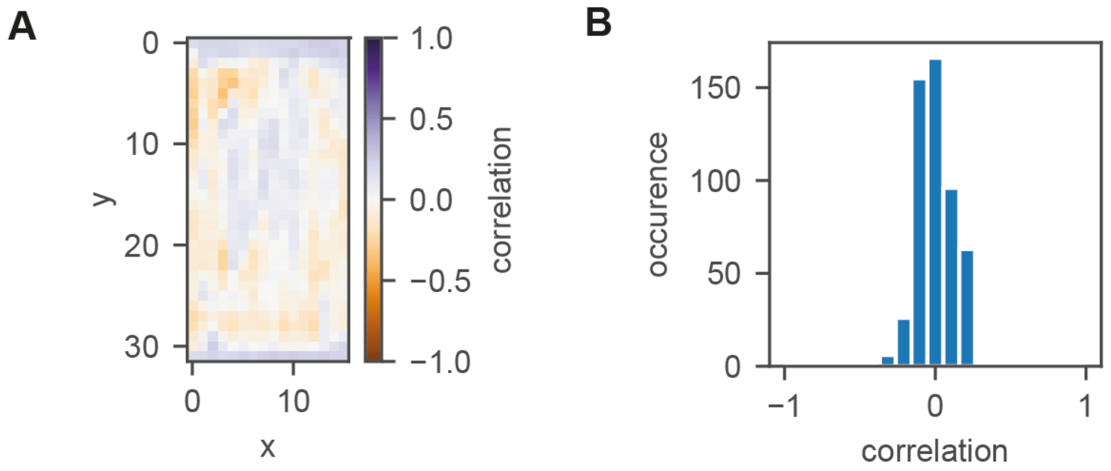

Supplementary Figure S3. Latent space pixels are not directly correlated with the glottal area. A: Latent space image Ψ_1 correlation with the segmented area. B: Distribution of correlation values.

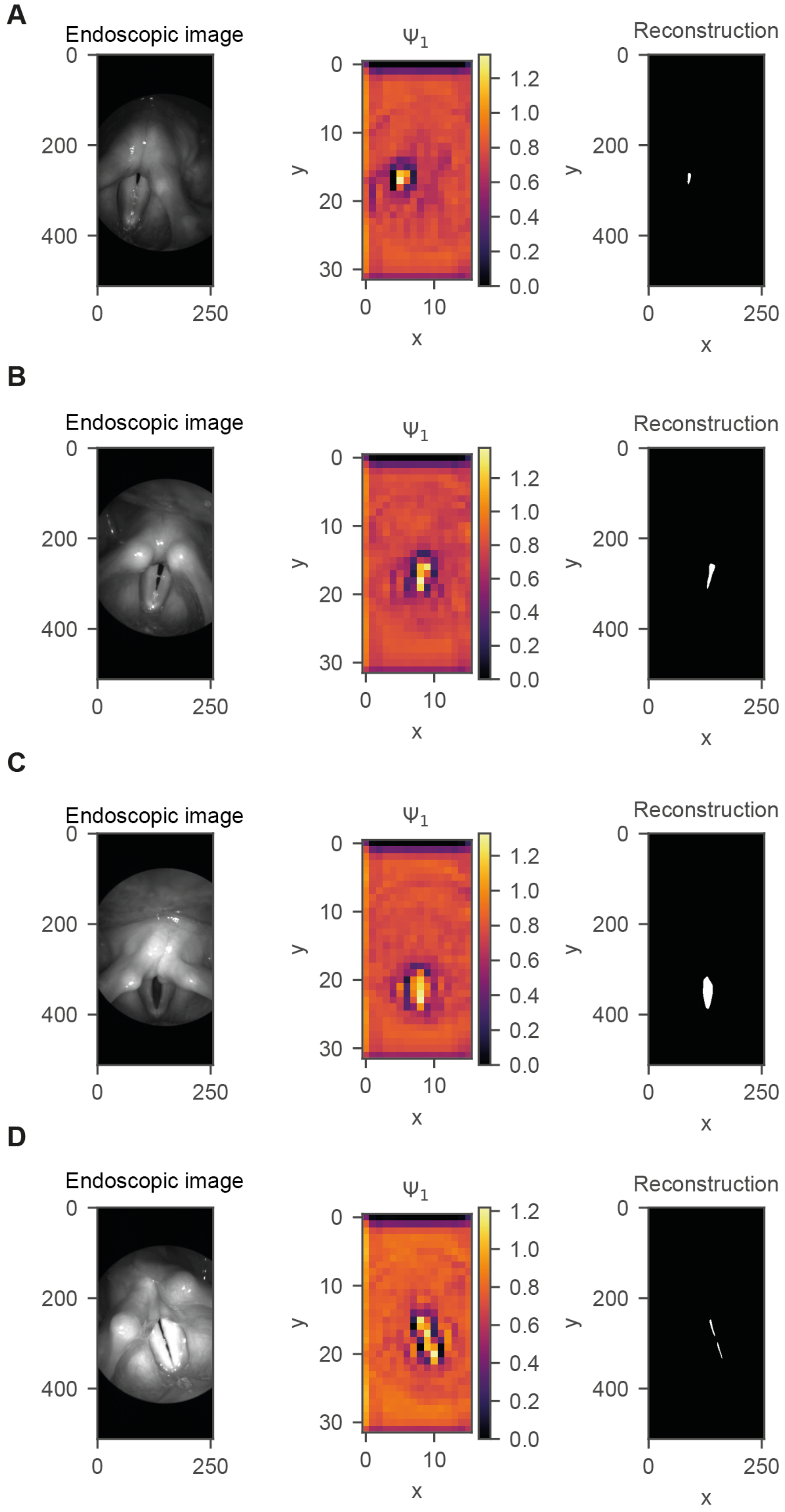

Supplementary Figure S4. Latent space coding is consistent across recordings.
A-D: Individual examples with endoscopic image, the corresponding latent space image $\Psi_{1,}$ and its reconstruction.

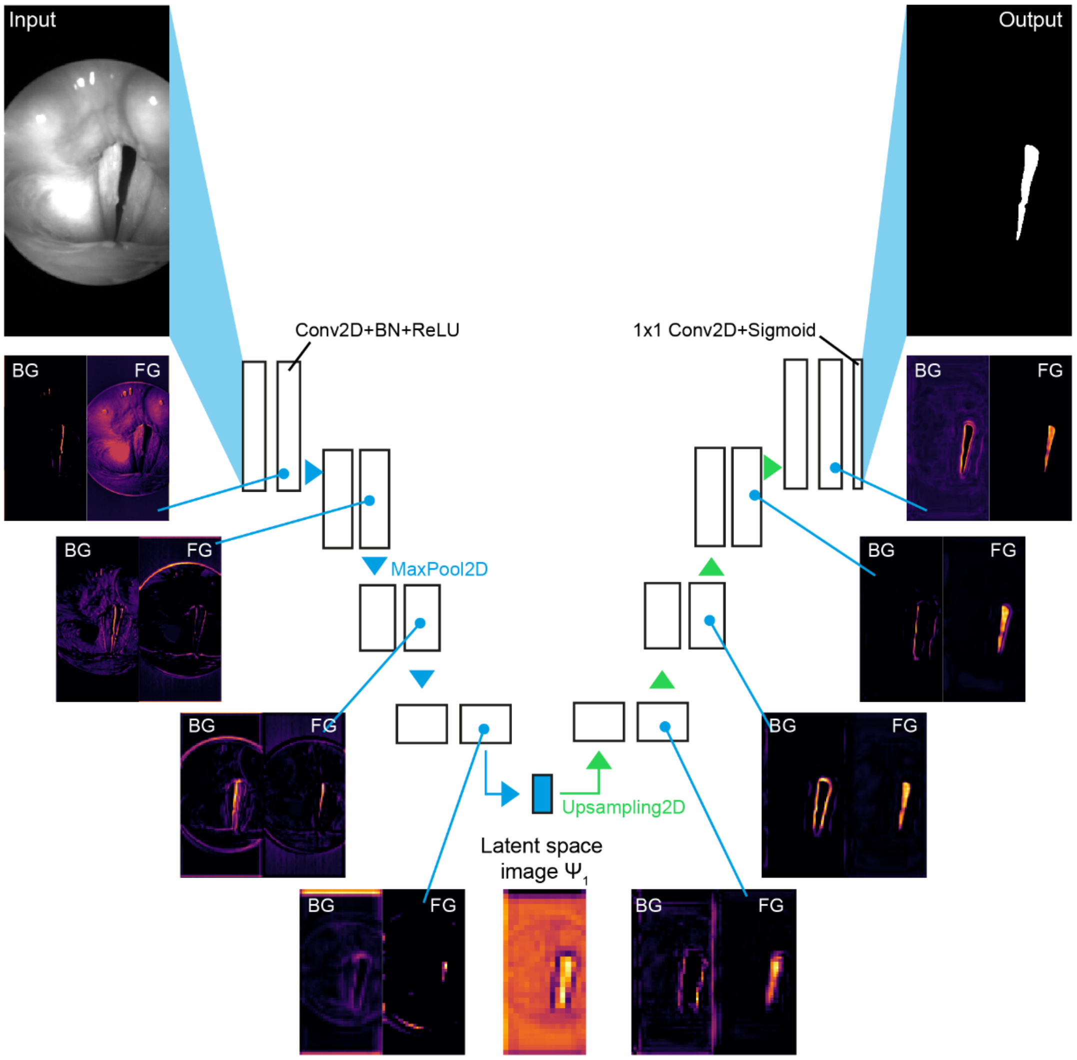

Supplementary Figure S5. Class activation maps show how the latent space is related to the input image. We show the class activation maps (CAMs) for the foreground (FG, glottal area) and the background (BG) at each layer in the U-Net architecture for the second convolutional layer. Note the focus on the glottal edges for the BG CAMs and the early emergence of the FG decoding.
